# Deficiencies in the Recognition and Reporting of Chronic Kidney Disease in Patients With Type 2 Diabetes Mellitus; A Hungarian Nationwide Analysis

**DOI:** 10.3389/ijph.2023.1606151

**Published:** 2023-08-29

**Authors:** Erzsébet Ladányi, Balázs Salfer, József Balla, István Kárpáti, György Reusz, Lilla Szabó, Péter Andriska, László Németh, István Wittmann, Boglárka Laczy

**Affiliations:** ^1^ Fresenius Medical Care Nephrology Center, Miskolc, Hungary; ^2^ AstraZeneca Ltd., Budapest, Hungary; ^3^ Department of Nephrology, Institute of Internal Medicine, Faculty of Medicine, University of Debrecen, Debrecen, Hungary; ^4^ First Department of Pediatrics, Semmelweis University, Budapest, Hungary; ^5^ Healthware Consulting Ltd., Budapest, Hungary; ^6^ Second Department of Medicine and Nephrology-Diabetes Center, University of Pécs Medical School, Pécs, Hungary

**Keywords:** chronic kidney disease, type 2 diabetes mellitus, prevalence, under-reporting, Hungary

## Abstract

**Objectives:** Recognition of chronic kidney disease (CKD) is crucial in type 2 diabetes mellitus (T2DM). We conducted a nationwide epidemiological study to evaluate T2DM-associated CKD in Hungary between 2016 and 2020.

**Methods:** Annual incidence and prevalence rates of registered CKD amongst all pharmacologically treated T2DM patients were analyzed in different age-groups by the central database of the Hungarian Health Insurance Fund Management. Statistical methods included Poisson regression, Bonferroni test, Chi-square test.

**Results:** We found 499,029 T2DM patients and 48,902 CKD patients in 2016, and 586,075 T2DM patients and 38,347 CKD patients in 2020. The majority of all prevalent T2DM and CKD patients were older (aged 60–69 years: 34.1% and 25.8%; ≥70 years: 36.1% and 64.4%, respectively). The annual incidence of T2DM and incidence rates of CKD in T2DM decreased in 2017–2020 (*p* < 0.001). The annual prevalence of T2DM increased (*p* < 0.01), the prevalence rates of CKD in T2DM were low and decreased from 9.8% to 6.5% in 2016–2020 (*p* < 0.001).

**Conclusion:** Incidence and prevalence of T2DM-associated CKD decreased significantly in Hungary in 2016–2020. Lower prevalence rates of CKD may suggest under-recognition and/or under-reporting.

## Introduction

Chronic kidney disease (CKD) has globally emerged as an increasing public health problem. CKD affects 15%–20% of the adult population worldwide, with slightly higher estimates in females, and one-third of those over 65 years [1–4]. The global prevalence and incidence of CKD, together with higher disability and death rates, have risen dramatically over the past three decades, largely driven by population growth and aging [1, 2]. The health and socioeconomic impact of CKD and type 2 diabetes mellitus (DM), which are often interrelated, is heavily increasing in all regions of the world, mainly due to the rising global burden of their common risk factors, such as the aging [1–3, 5–7].

CKD is associated with an excess risk of cardiovascular disease (CVD), end-stage kidney disease (ESKD), hospitalizations, and mortality [8–12], all of which are accelerated in the co-presence of DM [13–16].

There is a bidirectional relationship between CKD and DM. Firstly, CKD due to diabetes is one of the most common microvascular complications of DM, affecting 20%–40% of all diabetics [5]. Diabetic kidney disease (>90% type 2 DM) has become the leading etiology of CKD worldwide, and thus the primary global cause of ESKD [1, 5–7], as a consequence of increasing DM prevalence [17]. Secondly, patients with CKD have a higher risk of developing new-onset DM, via increased insulin resistance associated with CKD [18–20]. CKD was shown to be an independent predictor of incident DM [18]. The incidence rate of type 2 DM is markedly higher amongst CKD patients, with further increases in ESKD in the general population [19]. The incidence of type 2 DM was found to be 1.51-fold higher in CKD compared to non-CKD subjects [20]. Therefore, as type 2 DM develops more frequently in CKD patients, some of them may have a combination of both non-diabetic CKD and diabetic kidney disease.

The comorbidity burden of CKD and DM, either as an etiological factor or associating condition, is related to poorer outcomes in all stages, by the multiplied risk for disability, incidence of ESKD, CVD morbidity, and premature mortality [1, 7, 13–16].

Early identification and timely prompt treatment of CKD in type 2 DM patients is of important clinical relevance [6, 21–23]; especially, in the light of recently available effective therapies to reduce ESKD progression and CV mortality [21, 24–26]. Evidence-based cardiorenoprotective drug therapy should include renin-angiotensin system (RAS) inhibitors, mineralocorticoid receptor antagonists (MRA), sodium-glucose cotransporter-2 (SGLT-2) inhibitors, and glucagon-like peptide-1 (GLP-1) analogues [21–24, 26]. The combination of drugs may have additive benefits to convey kidney and CV protection, for example, as it was reported for the dual use of SGLT2-inhibitor (dapagliflozin) and MRA (eplerenone) in early CKD stages with albuminuria [25]. Additionally, dapagliflozin reduced by one-third the incidence of new-onset type 2 DM in patients with CKD or heart failure [27].

CKD in type 2 DM patients is clinically defined as a persistent decline in the estimated glomerular filtration rate (eGFR <60 mL/min/1.73 m^2^) and/or elevated urinary albumin excretion (UAE ≥30 mg/g) by the KDIGO (Kidney Disease: Improving Global Outcomes) [22]. Screening for CKD in type 2 DM patients, from the time of the diagnosis, is clearly recommended by regularly assessing eGFR and albuminuria [23, 26]. Furthermore, novel biomarkers may be useful tools for clinicians to detect CKD earlier, mainly in asymptomatic stages, also to specify individual patient’s CV risks and CVDs (e.g., heart failure, coronary heart disease) in advance, and to predict those who respond better to a specific therapy [28].

The prevalence of type 2 DM-associated CKD was 43.5% in US adults, based on the National Health and Nutrition Examination Survey (NHANES, 1999–2012) [29]. Prevalence estimations for CKD in type 2 DM were very similar in a large number of other reports from Europe and many other parts of the world [16, 30–36], showing that approximately 20% of type 2 DM patients exhibited lower eGFR (<60 mL/min/1.73 m^2^) and 30%–50% had elevated UAE level, despite the methodological differences regarding the definition, study settings, population under study, and data sources.

Unfortunately, in Hungary there is no CKD registry, and nationwide epidemiological data of CKD are lacking. We have recently reported regional prevalence data of CKD in a Hungarian subpopulation, with a total prevalence of 12.5% standardized by age and sex, where only 28.6% of laboratory-confirmed CKD patients were diagnosis-coded [37]. Although a series of nationwide registry-based analyses to evaluate the long-term epidemiological changes of type 2 DM have been conducted until 2016, none of these had CKD-related data [38–40]. To fill this substantial gap, we designed to obtain nationwide epidemiological data of CKD in high-risk type 2 DM patients.

The objective of the present nationwide CKD-EPI-HUN (Chronic Kidney Disease Epidemiology in Hungary) study was to determine the changes of annual incidence and prevalence of type 2 DM, CKD, and CKD amongst type 2 DM patients (aged >18 years) by age- and sex-specific manner in Hungary during the study period of 2016 and 2020 (for prevalence in 2016–2020, and for incidence in 2017–2020), using the central registry of the National Health Insurance Fund Management (NHIFM). We compared, by age-groups, the prevalence data of type 2 DM, CKD, and CKD in type 2 DM between 2016 and 2020. Data were collected to identify CKD based on diagnosis codes, thus reporting the tendency of CKD in the high-risk patients with type 2 DM could be also explored.

## Methods

### Study Design

In this nationwide, descriptive epidemiological study, we retrospectively analyzed longitudinal data from the Hungarian NHIFM database (license number: I043/72-6/2020). All data were extracted anonymously, remained unidentifiable for further analyses at the patient level, and were presented as aggregated output results, in alignment with the NHIFM data protection policy. The study protocol was reviewed and approved by the Ethics Committee of the University of Pécs (approval number: 9005-PTE 2022).

In Hungary, healthcare-related services and expenses are covered by insurance via the social security system, and because basic health insurance is obligatory for all residents, the NHIFM database encompasses almost 100% of the Hungarian population (8,003,000 adult subjects in 2020 by the Central Statistical Office [41]). Therefore, our study could be considered a nationwide one.

Health claims data of all medical procedures, reimbursements and pharmacy dispensed prescriptions, with the assigned social security number (i.e., patient), are regularly registered in the NHIFM database. This central registry includes records of the patients’ social security number, year of birth, sex, postal address code, ICD (International Classification of Diseases, 10th version) codes, ICHI (International Classification of Health Intervention) codes, and the level of health service (e.g., outpatient, inpatient). Although our healthcare system involves gatekeeping structure, where patients can easily get access to the primary care providers, referral is required for the hospital and ambulatory specialist care.

Data from all relevant financing registers, outpatient and inpatient care data, redemptions of drug prescriptions with reimbursement, renal replacement treatment data were collected in this study. Of note, nearly all anti-diabetic drugs are subjected to reimbursement with different percentage (50%–100%) in Hungary (except for one formulation of metformin) [39], and reimbursements of these drugs were not changed during the study period.

### Identification of Patients

We examined Hungarian adults (over ≥18 years) who had a social security number, thus analyzable data in the NHIFM registry, and had DM-specific ICD codes of E10/E11/E14 (either as a primary or secondary diagnosis) via outpatient or inpatient occurrence. Among this entire population of people with DM, we included those who had pharmacy redeemed prescription of an anti-diabetic medication (oral drugs, non-insulin injections, insulin; ATC A10 class) at least one occasion during the period from 1st of January 2016 to 31st of December 2020.

The method for the identification and classification of DM patients was systematically and fully described earlier [38], and was used in subsequent publications [39, 40].

Exclusion criteria were as follows: i) patients who had no ICD codes of E10/E11/E14, and redeemed the anti-diabetic drug less than 3 times, and did not die within 120 days after the anti-diabetic drug redemption, and did not reach the end of the study period within 120 days after the last anti-diabetic drug redemption; or ii) when redemption was later than the month of death [38–40].

Women with gestational diabetes (ICD-10: O24.4), and those with polycystic ovary syndrome (ICD-10: E28.2), if they were reported by these diagnosis codes at least once during the study period, were also excluded from the analysis [38–40].

Patients with type 1 DM (ICD-10: E10) were also excluded, based on a hierarchical system consisting of one basic and five more stepwise definitions [38–40]. The basic definition included the following criteria: patient had E10 code; and the E10/(E10 + E11) code ratio was ≥50%; and dispensed insulin prescription; and had no oral anti-diabetic drug dispense for 180 days prior to the first anti-diabetic drug redemption [38–40]. Type 1 DM was established when the basic definition or the first hierarchical supplementary condition was fulfilled [38–40].

Subsequently, those patients who did not qualify as having type 1 DM according to these criteria were considered to have type 2 DM (ICD-10: E11), and they were all enrolled in this present study. Thus, we included all patients who were treated pharmacologically for type 2 DM.

DM was established from the day when any of the following criteria was first fulfilled: redemption of anti-diabetic drug, or E10/E11/E14 code occurred during outpatient or inpatient care, followed by another diagnosis code (E10, E11, E14) over 30 days but within 180 days, or the patient died within 60 days [38–40].

CKD patients were then identified within the population of enrolled type 2 DM patients. CKD patients were defined as having ICD codes for chronic renal impairment, including chronic renal failure (ICD-10: N18), or unspecified renal failure (ICD-10: N19) during the period from 1st of January 2016 to 31st of December 2020. CKD was diagnosed on the date when the patient was first registered, taking into account the chronicity by having at least two separate ICD codes within the entire study period.

### Incidence

Incidence was defined as the number of newly registered type 2 DM and CKD patients, based on their first analyzable report. New cases were counted for each calendar year (i.e., 1st January to 31st December).

Annual incidence, as the annual number of newly registered type 2 DM and CKD patients, was expressed as crude numbers (N).

We used the incidence rate to determine the ratio of new CKD patients amongst new type 2 DM patients, by expressing the number of incident CKD patients relative to the number of incident type 2 DM patients in percentage.

The number of incident patients in 2016, the first study year, does not reveal merely the newly diagnosed cases, as per definition it designates the first time when the patient received healthcare due to type 2 DM or CKD, and thus it may involve earlier diagnosed cases based on dual ICD code criteria.

Therefore, in order to detect consistently the real new cases, incidence data of DM and CKD patients were calculated from 2017 to 2020.

### Prevalence

Prevalence was defined as the total annual number of patients with type 2 DM and CKD and was counted for each calendar year (i.e., 1st January to 31st December).

Annual prevalence data included the number of prevalent patients, who were alive on 1st of January in the given year (with previous registration in the database, so the first year of incidence was in preceding years), and the number of newly registered patients in the corresponding entire year (the first year of incidence was in the same year).

Annual prevalence, as the annual number of all prevalent patients with type 2 DM and CKD, was expressed as crude numbers (N).

The prevalence rate was used to determine the ratio of all prevalent CKD patients amongst all prevalent type 2 DM patients, expressed in percentage.

We also demonstrate the demographic features, providing sex (male/female) and age distribution data (in age-groups of <20, 20–29, 30–39, 40–49, 50–59, 60–69, and ≥70 years) of all prevalent DM and CKD patients between 2016 and 2020.

### Statistical Analysis

Trends of the annual changes for the incidence and prevalence of type 2 DM and CKD were analyzed by Poisson regression. Trends by age-groups for the prevalence of type 2 DM and CKD were tested by Poisson regression, and corresponding *p*-values of age-groups were adjusted with Bonferroni test. Chi-square test was used to compare the proportions of prevalent type 2 DM and CKD patients by age distribution. Proportion test was used to compare the ratios of CKD in type 2 DM by age distribution. All analyses were performed by R programming language (version 4.0.4.). *p* < 0.05 was considered statistically significant.

## Results

### Identification of Patients

**FIGURE 1:**
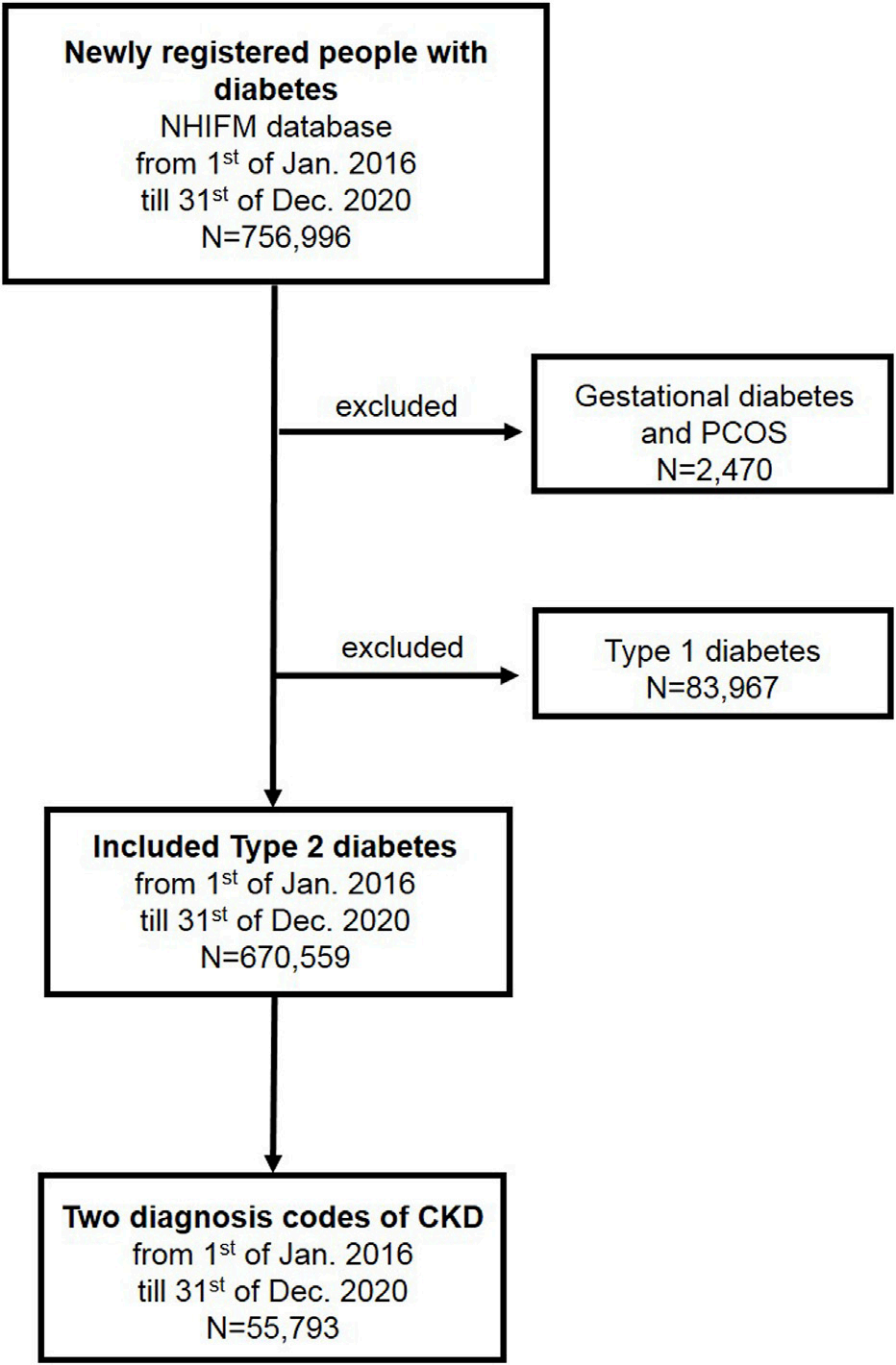
Patient-flow diagram. (Deficiencies in the recognition and reporting of chronic kidney disease in patients with type 2 diabetes mellitus, Hungary, 2016–2020).

The population of subjects with newly registered diabetes totaled 756,996 patients in Hungary during the study period of 2016 and 2020. After excluding those with gestational diabetes and polycystic ovary syndrome (N = 2,470) and type 1 DM (N = 83,967), there were 670,559 patients with type 2 DM who were included in the study (Figure 1). Amongst these type 2 DM patients, the total number of patients who met the ICD-based criteria for CKD was 55,793 during the study period of 2016 and 2020 (Figure 1).

### Incidence

Incidence data of type 2 DM and CKD patients were analyzed from 2017 to attain consistent and real data of newly reported cases in the registry, hence 2016 was omitted, based on the definitions described in the methodology.

**FIGURE 2:**
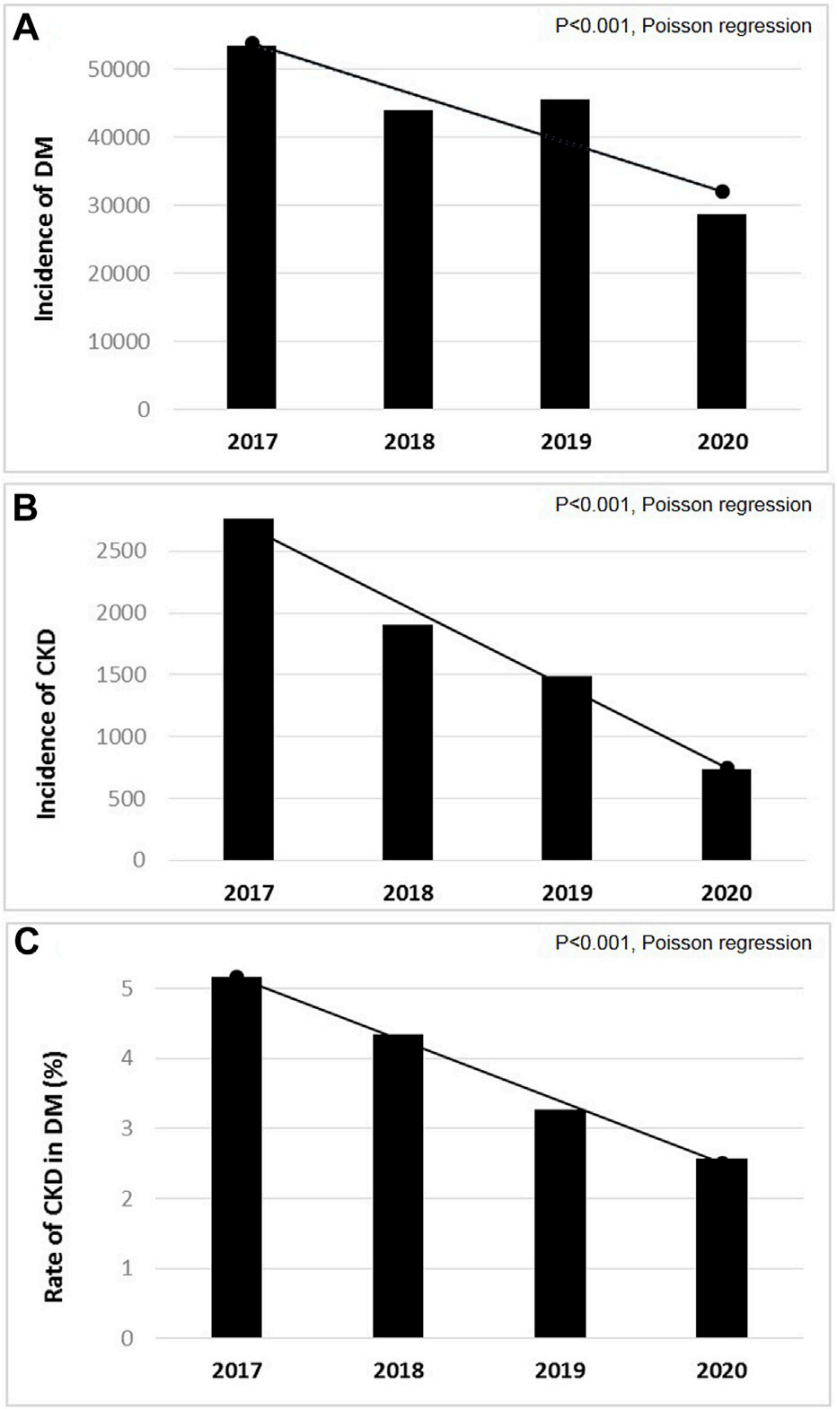
The annual incidence of patients with **(A)** pharmacologically treated type 2 diabetes mellitus (DM); and **(B)** chronic kidney disease (CKD) amongst them; and **(C)** incidence rates of CKD in type 2 DM between 2017 and 2020 in Hungary (*p* < 0.001, Poisson regression). (Deficiencies in the recognition and reporting of chronic kidney disease in patients with type 2 diabetes mellitus, Hungary, 2016–2020).

The number of incident type 2 DM patients decreased markedly (from 53,398 to 28,765 cases) between 2017 and 2020 (*p* < 0.001, Figure 2A).

The number of incident CKD patients decreased gradually (from 2,759 to 740 cases) between 2017 and 2020 (*p* < 0.001, Figure 2B).

The annual incidence rates of CKD in type 2 DM decreased from 5.2% to 2.6% between 2017 and 2020, which was significant by trend analysis (*p* < 0.001, Figure 2C).

### Prevalence

The prevalence of type 2 DM increased gradually (from 499,029 to 586,075 cases) between 2016 and 2020 (Table 1), at a relatively constant yearly rate, which was lessened in 2020. The increasing annual type 2 DM prevalence was significant by trend analysis in the periods of 2016–2020 and 2016–2019 (*p* < 0.01). There was female dominance among type 2 DM patients in each study year, 52.8% (263,297 cases) in 2016, and 52.3% (306,231 cases) in 2020 were females (Table 1).

**TABLE 1:** The number of patients with pharmacologically treated type 2 diabetes mellitus, and with chronic kidney disease amongst them between 2016 and 2020 in Hungary. (Deficiencies in the recognition and reporting of chronic kidney disease in patients with type 2 diabetes mellitus, Hungary, 2016–2020).

Study year	DM prevalence		CKD prevalence in DM	
	Male/Female	Total (N)	Male/Female	Total (N)
2016	235,732/263,297	499,029	21,339/27,563	48,902
2017	254,430/282,658	537,088	21,397/27,899	49,296
2018	265,501/293,981	559,482	20,140/26,687	46,827
2019	277,041/304,684	581,725	18,497/24,764	43,261
2020	279,844/306,231	586,075	16,328/22,019	38,347

DM, type 2 diabetes mellitus; CKD, chronic kidney disease.

**FIGURE 3:**
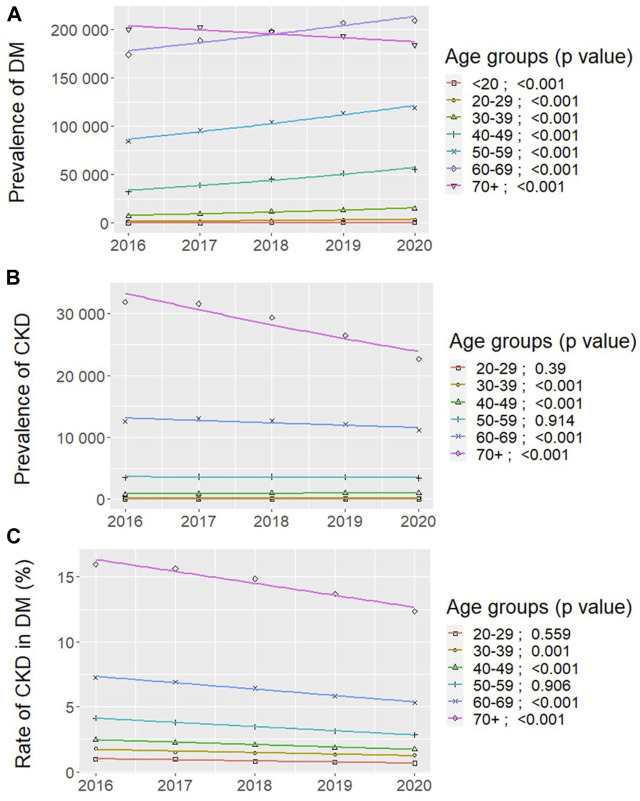
Changes by age-groups in the distribution of all prevalent patients with **(A)** pharmacologically treated type 2 diabetes mellitus (DM); and **(B)** chronic kidney disease (CKD) amongst them; and **(C)** prevalence rates of CKD in type 2 DM between 2016 and 2020 in Hungary (Bonferroni-adjusted *p* values, Poisson regression). (Deficiencies in the recognition and reporting of chronic kidney disease in patients with type 2 diabetes mellitus, Hungary, 2016–2020).

The prevalence of type 2 DM was higher in older ages in each study year (Figure 3A; Supplementary Table S1). The majority of all prevalent type 2 DM patients for the total period of 2016–2020 was observed in the 60–69 years (34.1%) and ≥70 years (36.1%) age-groups (Supplementary Table S1).

The number of prevalent type 2 DM patients clearly increased in all age-groups between 2016 and 2020 (*p* < 0.001, Figure 3A), except in the ≥70 years age-group, where it significantly decreased (from 199,698 to 183,554 cases) (*p* < 0.001, Figure 3A; Supplementary Table S1).

The age distribution of prevalent type 2 DM patients differed significantly in 2016 and 2020 (Supplementary Figure S1), where the proportion of patients decreased only in the ≥70 years age-group (from 40.0% to 31.3%), while it increased in all other younger (<20–69 years) decades (*p* < 0.001, Supplementary Figure S1).

The prevalence of CKD decreased (from 48,902 to 38,347 cases) between 2016 and 2020, and was the lowest in 2020 (*p* < 0.05, Table 1). There were more women among CKD patients in each study year, we found 27,563 (56.3%) females in 2016, and 22,019 (57.4%) females in 2020 (Table 1).

The prevalence of CKD was higher in older ages in each study year (Figure 3B; Supplementary Table S2). The largest part of all prevalent CKD patients for the total period of 2016–2020 was within those over 70 years of age (64.4%), followed by the age-group of 60–69 years (25.8%) (Supplementary Table S2).

The number of prevalent CKD patients showed distinct changes in different age-categories (Figure 3B). According to the NHIFM reporting principles, sections with less than 10 patients could not be extracted, therefore data of the <20 years age-group are not presented. The number of prevalent CKD patients increased in the 30–39 years and 40–49 years age-groups between 2016 and 2020 (*p* < 0.001, Figure 3B; Supplementary Table S2), and remained unaltered in the 50–59 years age-group. In contrast, the number of prevalent CKD patients decreased steadily in those over 60 years of age (*p* < 0.001, Figure 3B; Supplementary Table S2).

The age distribution of prevalent CKD patients changed significantly between 2016 and 2020 (Supplementary Figure S2), as the proportion of patients decreased in the ≥70 years age-group (from 65.2% to 59.1%), while it increased in patients with 20–69 years of age (*p* < 0.001, Supplementary Figure S2).

The prevalence rates of CKD in type 2 DM were consistently higher with older age in each study year (Figure 3C), with the highest ratio in the >70 years age-group (14.85%) for the entire period of 2016–2020 (Figure 3C; Supplementary Table S3).

The prevalence rates of CKD in type 2 DM were numerically decreasing in all age-categories between 2016 and 2020 (Figure 3C; Supplementary Table S3), however, reductions were significant in the age-groups of 30–39 years, 40–49 years (*p* ≤ 0.001), where both the number of prevalent type 2 DM and CKD patients were significantly increased (Figures 3A,B). Reduced prevalence rates of CKD in type 2 DM were also significant in the age-groups of 60–69 years and >70 years (*p* < 0.001, Figure 3C).

The net difference in the prevalence rate of CKD in type 2 DM by age distribution was not significant between 2016 and 2020 (Supplementary Figure S3).

**FIGURE 4:**
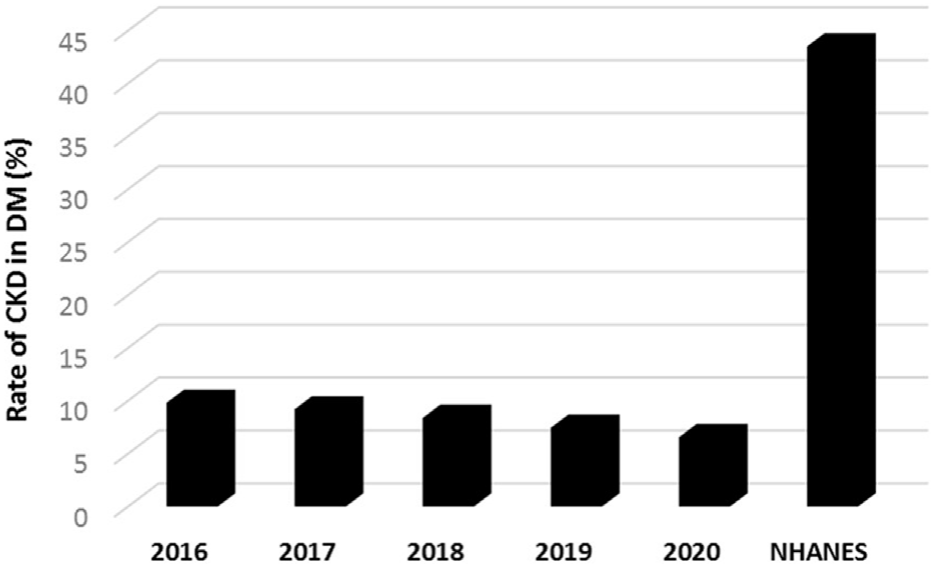
The annual prevalence rates of chronic kidney disease (CKD) in patients with pharmacologically treated type 2 diabetes mellitus (DM) between 2016 and 2020 in Hungary (*p* < 0.001, for trend by Poisson regression); and compared to the prevalence estimates of the National Health and Nutrition Examination Survey (NHANES, 1999–2012) based on data in reference 29. (Deficiencies in the recognition and reporting of chronic kidney disease in patients with type 2 diabetes mellitus, Hungary, 2016–2020).

The prevalence rate of CKD in type 2 DM decreased gradually from 9.8% to 6.5% between 2016 and 2020 (*p* < 0.001, Figure 4; Supplementary Table S3).

The most acknowledged and cited prevalence rate of CKD amongst type 2 DM patients was 43.5%, reported by the NHANES (1999–2012) in the US population [29]. In comparison, our results showed substantially lower prevalence rates of CKD in type 2 DM (ranged between 9.8% and 5.6%), which totaled 8.3% including all patients during the entire study period of 2016–2020 (Figure 4).

## Discussion

This nationwide CKD-EPI-HUN study is the first to report epidemiological data of type 2 DM-associated CKD in Hungary. The key findings of our study were as follows: i) the annual incidence of type 2 DM and associated CKD decreased significantly in 2017–2020; ii) the majority of all prevalent type 2 DM (70%) and CKD patients (90%) were older than 60 years; iii) the annual prevalence of type 2 DM increased significantly in 2016–2020; iv) the prevalence rates of type 2 DM-associated CKD were low and decreased significantly in 2016–2020, suggesting under-reporting.

Decreasing incidence of type 2 DM in Hungary was demonstrated in the preceding period from 2001 to 2016, in all decades over 30 years of age [39]. Other reports also indicated a decreasing incidence of type 2 DM, including a recent analysis of 24 population-based datasets showing decreasing trends from 2010 onwards in most high-income countries [42]. The age-adjusted incidence of DM was shown to decrease in US adults after 2008 [43]. Our data that showed decreasing incidence of type 2 DM, seem consistent with these studies.

The decline in 2020 could be caused, in part, by deterrent effects of the COVID-19 pandemic, rather than a true decrease in the number of new DM cases. The COVID-19 outburst in 2020 might have contributed to the lower number of new DM cases, as deferred visits of patients and personal access to healthcare services had generally decreased.

By the Global Disease Burden study, the incidence of type 2 DM-related CKD has increased worldwide between 1990 and 2019, in both sexes and with a peak incidence in people at age of 80 years [7]. In this study the age-standardized incidence rates of CKD due to type 2 DM have increased in Europe (with an estimated annual percentage change (EAPC) of 0.82, 95% CI: 0.79–0.84), and an increase was also estimated for Hungary (EAPC: 1.99, 95% CI: 1.86–2.12), but it remained stable, for example, in the US (EAPC: 0.09, 95% CI: −0.01–0.18) [7].

As opposed to the anticipated increases, we found that the incidence rate of CKD in type 2 DM decreased gradually and significantly from 5.2% to 2.6% between 2017 and 2020. The very low number of new cases in 2020 (which was less by 73% vs. 2017 and by 50% vs. 2019) could be attributed to the under-detection of CKD patients during the COVID-19 outbreak. However, prior to 2020, the incidence rate of CKD also significantly decreased in 2017–2019.

The prevalence of type 2 DM showed an upward trend. This was also reported to increase from 2001 to 2016, although it plateaued between 2011 and 2016 in Hungary [39]. The global prevalence of DM is known to progressively increase annually [17]. In the US, the age-adjusted prevalence of diagnosed DM markedly increased during the period of 2001–2020 [43]. The decreasing incidence rates, together with increasing prevalence rates suggest an improved survival of type 2 DM patients, possibly due to the decreasing mortality, which was documented for type 2 DM patients in Hungary, including those over 60 years of age [40].

The prevalence of DM increases with aging, covering 20%–25% of patients over 65 years of age [17, 39, 43]. Consistently, we found higher percentage of prevalent type 2 DM patients with older age, reaching 34.1% and 36.1% among those aged 60–69 years and ≥70 years, respectively. In these elderly, there is a higher rate of background CKD, even in the absence of type 2 DM, affecting one-third of the general population aged over 65 years [1–4]. In type 2 DM, CKD affects about 30%–50% of the patients [16, 30–36], and the prevalence may be higher (about 60%) in those over 65 years of age [29]. Here we also found that prevalence of CKD in type 2 DM was higher in older ages, and majority of patients were in the 60–69 years (25.8%) and ≥70 years (64.4%) age-groups.

We found that the prevalence rate of CKD in type 2 DM decreased gradually and significantly from 9.8% to 6.5% between 2016 and 2020, and the decline was present in most of the age-categories. The impact of the COVID-19 pandemic in 2020 cannot be excluded, however, the prevalence of type 2 DM increased in 2016–2020, whilst the prevalence of CKD decreased in 2016–2019. Alternatively, the increasing use of novel effective anti-diabetic therapies (e.g., SGLT-2 inhibitors, GLP-1 analogues) could also have benefits on kidney complications [21, 23–25], however, it is unlikely to explain the very low number of identified CKD cases after such a short-term of their clinical application.

A real decrease in the CKD prevalence is not likely; our results rather suggest that CKD was underdiagnosed. Although awareness of CKD usually improves in older ages by more frequent eGFR measurements, especially in DM where CKD screening is annually recommended [21–23, 26].

As the DM prevalence increases, relatively more patients will be affected by CKD. Indeed, the prevalence of diabetic kidney disease in the US increased proportionally with the prevalence of DM (NHANES, 1988–2008) [44]. The prevalence of CKD amongst type 2 DM patients was 43.5% in the US population using the KDIGO definitions (NHANES, 1999–2012) [29]. The Global Disease Burden study indicated that age-standardized prevalence rates of CKD due to type 2 DM increased between 1990 and 2019 in most countries, including the US (EAPC: 0.16, 95% CI: 0.1–0.21), and Hungary (EAPC: 0.2, 95% CI: 0.17–0.23) [7]. For Hungary, the number of prevalent type 2 DM-associated CKD patients was estimated 224,771 (95% UI: 206,459–244,932) in 2019 [7]. Given the number of registered type 2 DM patients in this study, by calculating with about 40% prevalence rate [4, 29, 45, 46], the number of individuals with CKD in type 2DM would be projected as 230,000 in Hungary, which approximated the international estimation [7]. In contrast, we found that the number of CKD cases in type 2 DM ranged between 48,902 and 38,347 during the study period of 2016–2020, with corresponding prevalence rates of 9.8% and 5.6%, indicating that the prevalence of CKD in type 2 DM were 5-6-times lower as compared to the international data [7, 29]. Possible underestimation of our data due to methodological reasons cannot be ruled out (e.g., different data source, confirmed CKD by two ICD codes), however, if we take into consideration that ICD based diagnosis of CKD covers about one-third of laboratory-positive cases [37, 47, 48], our results still substantially lag behind the international prevalence estimations.

The under-reporting of CKD in the general population was documented in most countries [37, 47, 48]. Our results indicate lower awareness of CKD also in type 2 DM patients, which could be a result of the failure to screen, recognize, or report CKD. In the background, the lack of knowledge and adherence to clinical practice guidelines has been implicated [49–51]. Moreover, a large number of patients, even those with high-risk conditions or with sustained reduction of eGFR, were not coded for CKD [51–55]. Albuminuria and eGFR tests are required at least annually in type 2 DM patients [21–23, 26]. While the eGFR test is more often used, albuminuria screening is generally underutilized (∼50%) to detect CKD in type 2 DM patients [49–51]. In Hungary, the kidney function test is part of the routine clinical laboratory tests for DM patients and fully reimbursed for primary care providers (in contrast to the albuminuria test, which was not subject to financial refunding during the study period). Considering eGFR, as the major basic screening parameter of CKD, we think that inadequate recognition and report of CKD, rather than insufficient testing of type 2 DM patients was the case in this study.

The strengths of this study are the nationwide nature, the large volume of data from the central registry, including age- and sex distributions, under real-world conditions. Our study has also some limitations. We detected CKD based on diagnosis codes due to missing laboratory data of eGFR and albuminuria, which could underestimate the number of true cases. Although administrative data sources have a lower sensitivity in CKD research [37, 47, 48], even these unvalidated data could be valuable to describe epidemiological trends. We had no clinical data of patients regarding lifestyle habits (smoking), glycemic control, duration of DM, stages of CKD, among others, mortality was not examined. We cannot capture type 2 DM patients with lifestyle therapy.

In conclusion, this CKD-EPI-HUN study is the first to provide nationwide data on the epidemiology of type 2 DM-associated CKD in Hungary by database analysis. Both incidence and prevalence of CKD in type 2 DM were at low rates and decreased significantly between 2016 and 2020 in Hungary, suggesting that the recognition and/or reporting of CKD is relatively insufficient in our country. Our findings support the urgent need for the better appreciation and identification of CKD amongst patients with type 2 DM.
