# Supplementary material for: Deficiencies in the Recognition and Reporting of Chronic Kidney Disease in Patients With Type 2 Diabetes Mellitus; A Hungarian Nationwide Analysis
Source: Int J Public Health. 2023 Aug 29;68:1606151. doi: 10.3389/ijph.2023.1606151 (PMC10496514; doi:10.3389/ijph.2023.1606151)
Supplement: Supplementary file 1 [file DataSheet1.PDF]

Supplementary Material

Deficiencies in the Recognition and Reporting of Chronic Kidney Disease in Patients with Type 2 Diabetes Mellitus; A Hungarian nationwide analysis

Table S1. The number and distribution by age groups of all prevalent patients with pharmacologically treated type 2 diabetes mellitus between 2016 and 2020 in Hungary. Data are presented for each study year, and for the total study period of 2016-2020. (Deficiencies in the recognition and reporting of chronic kidney disease in patients with type 2 diabetes mellitus, Hungary, 2016-2020)

Study year	2016	2017	2018	2019	2020	2016-2020
Age group (years)	N (%)	N (%)	N (%)	N (%)	N (%)	N (%)
<20	205 (0.0)	297 (0.1)	397 (0.1)	488 (0.1)	557 (0.1)	559 (0.1)
20-29	1,442 (0.3)	2,003 (0.4)	2,537 (0.5)	3,139 (0.5)	3,673 (0.6)	3,696 (0.6)
30-39	7,436 (1.5)	9,611 (1.8)	11,517 (2.1)	13,459 (2.3)	15,007 (2.6)	15,141 (2.3)
40-49	32,235 (6.5)	39,294 (7.3)	45,260 (8.1)	51,264 (8.8)	55,564 (9.5)	56,635 (8.4)
50-59	84,124 (16.9)	95,549 (17.8)	104,501 (18.7)	113,431 (19.5)	118,693 (20.3)	124,263 (18.5)
60-69	173,889 (34.8)	188,314 (35.1)	197,711 (35.3)	206,583 (35.5)	209,027 (35.7)	228,354 (34.1)
70+	199,698 (40.0)	202,020 (37.6)	197,559 (35.3)	193,361 (33.2)	183,554 (31.3)	241,911 (36.1)

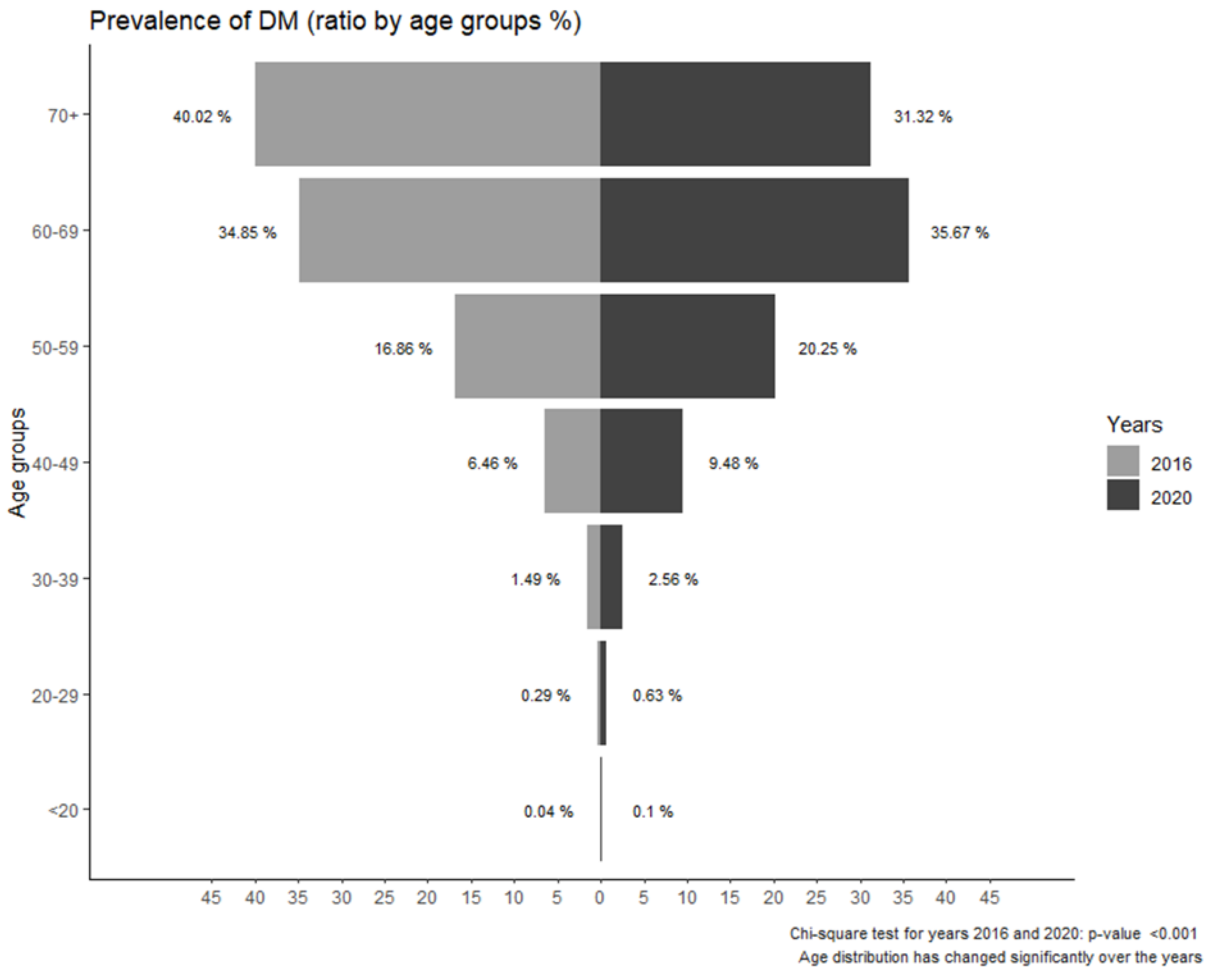

Figure S1. The proportion by age-groups of prevalent patients with pharmacologically treated type 2 diabetes mellitus (DM) between 2016 and 2020 in Hungary ($P<0.001$, Chi-square test). (Deficiencies in the recognition and reporting of chronic kidney disease in patients with type 2 diabetes mellitus, Hungary, 2016-2020)

Table S2. The number and distribution by age groups of all prevalent patients with chronic kidney disease amongst patients with pharmacologically treated type 2 diabetes mellitus between 2016 and 2020 in Hungary. Data are presented for each study year, and for the total study period of 2016-2020. (Deficiencies in the recognition and reporting of chronic kidney disease in patients with type 2 diabetes mellitus, Hungary, 2016-2020)

**Study year**	**2016**	**2017**	**2018**	**2019**	**2020**	**2016-2020**
**Age group (years)**	**N (%)**	**N (%)**	**N (%)**	**N (%)**	**N (%)**	**N (%)**
20-29	14 (0.0)	19 (0.0)	20 (0.0)	24 (0.1)	24 (0.1)	26 (0.0)
30-39	131 (0.3)	146 (0.3)	166 (0.4)	183 (0.4)	187 (0.5)	202 (0.4)
40-49	797 (1.6)	888 (1.8)	943 (2.0)	956 (2.2)	966 (2.5)	1,097 (2.0)
50-59	3,464 (7.1)	3,620 (7.3)	3,615 (7.7)	3,553 (8.2)	3,366 (8.8)	4,141 (7.4)
60-69	12,612 (25.8)	13,005 (26.4)	12,721 (27.2)	12,073 (27.9)	11,132 (29.0)	14,402 (25.8)
70+	31,883 (65.2)	31,617 (64.1)	29,361 (62.7)	26,470 (61.2)	22,670 (59.1)	35,922 (64.4)

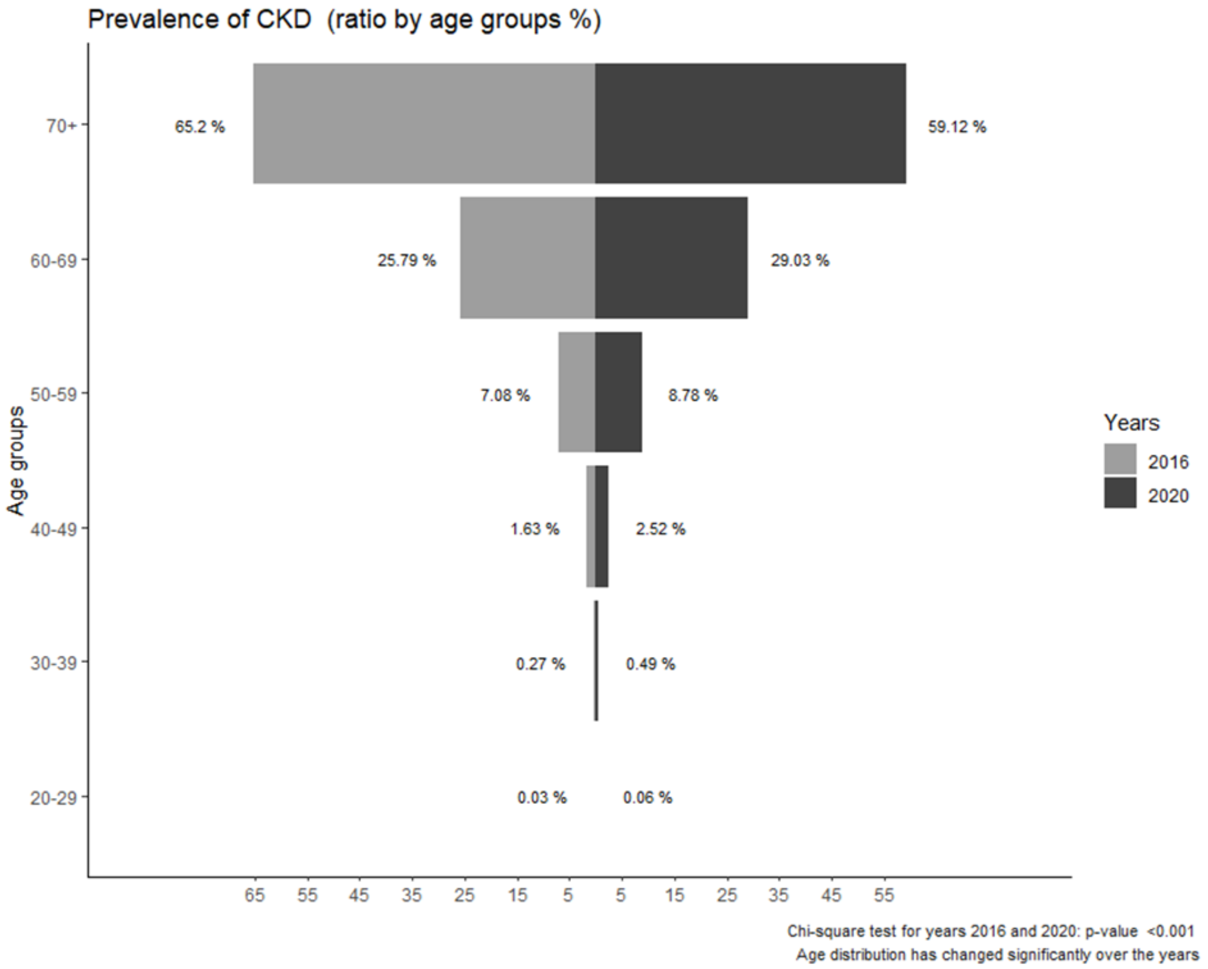

Figure S2. The proportion by age-groups of prevalent patients with chronic kidney disease (CKD) amongst patients with pharmacologically treated type 2 diabetes mellitus between 2016 and 2020 in Hungary (P<0.001, Chi-square test). (Deficiencies in the recognition and reporting of chronic kidney disease in patients with type 2 diabetes mellitus, Hungary, 2016-2020)

Table S3. Prevalence rates of chronic kidney disease in type 2 diabetes mellitus by age groups between 2016 and 2020 in Hungary. Data are presented for each study year, and for the total study period of 2016-2020. (Deficiencies in the recognition and reporting of chronic kidney disease in patients with type 2 diabetes mellitus, Hungary, 2016-2020)

Study year	2016	2017	2018	2019	2020	2016-2020
Age group (years)	(%)	(%)	(%)	(%)	(%)	(%)
20-29	0.97	0.95	0.79	0.76	0.65	0.70
30-39	1.76	1.52	1.44	1.36	1.25	1.33
40-49	2.47	2.26	2.08	1.86	1.74	1.94
50-59	4.12	3.79	3.46	3.13	2.84	3.33
60-69	7.25	6.91	6.43	5.84	5.33	6.31
70+	15.97	15.65	14.86	13.69	12.35	14.85
Total	9.80	9.18	8.37	7.44	6.54	8.32

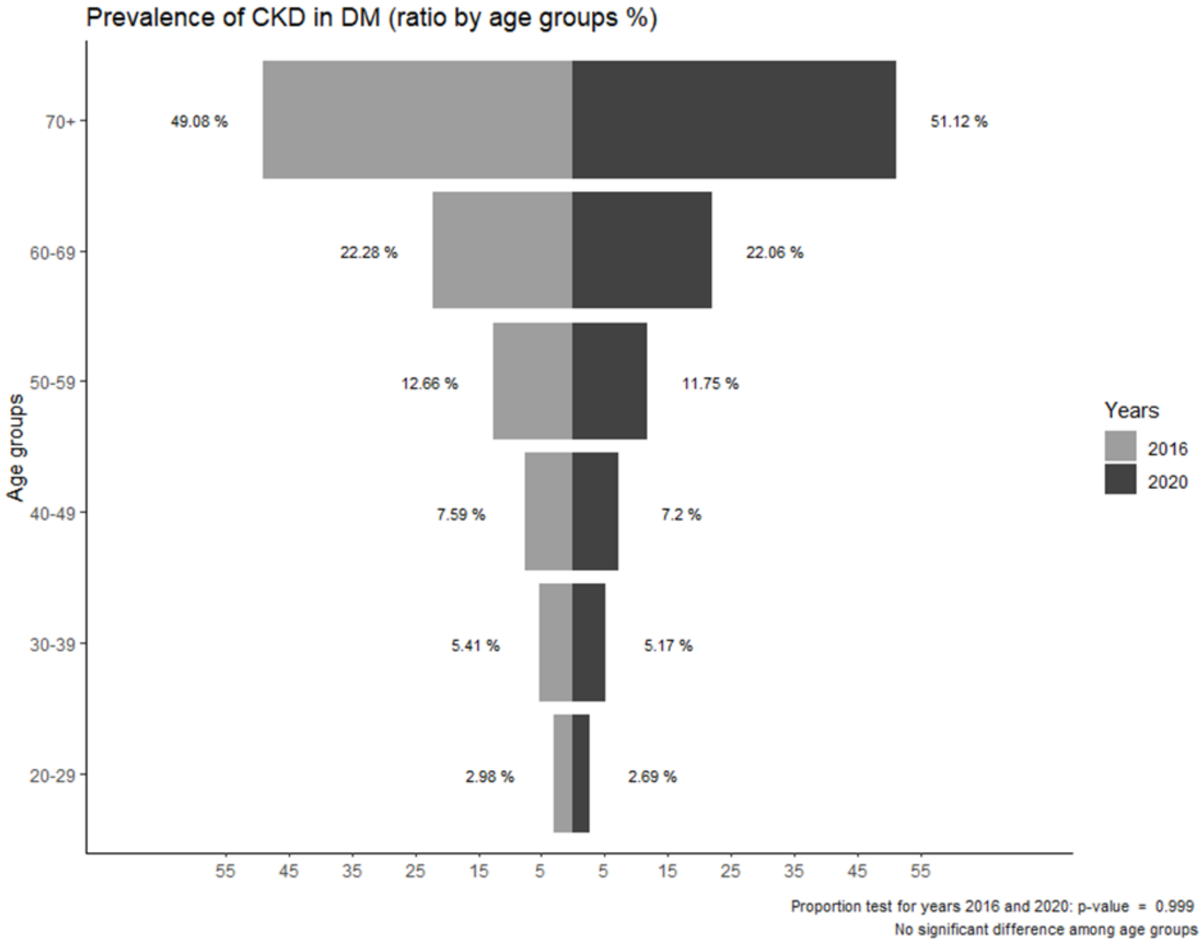

Figure S3. Age distribution of the prevalence rates of chronic kidney disease (CKD) in type 2 diabetes mellitus (DM) between 2016 and 2020 in Hungary (NS, Proportion test). (Deficiencies in the recognition and reporting of chronic kidney disease in patients with type 2 diabetes mellitus, Hungary, 2016-2020)
